# Franseen Needles May Be Promising for Improving the Sampling Adequacy of EUS-FNA for Subepithelial Lesions

**DOI:** 10.3390/diagnostics12071667

**Published:** 2022-07-09

**Authors:** Noriki Kasuga, Yusuke Kurita, Emiko Tanida, Shin Yagi, Ko Suzuki, Sho Hasegawa, Takamitsu Sato, Kunihiro Hosono, Shingo Kato, Yusuke Sekino, Noritoshi Kobayashi, Itaru Endo, Kensuke Kubota, Atsushi Nakajima

**Affiliations:** 1 Department of Gastroenterology and Hepatology, Yokohama City University Hospital, Yokohama 236-0004, Japan; kasuganoriki1992@gmail.com (N.K.); e103083b@gmail.com (S.Y.); kosuzuki@yokohama-cu.ac.jp (K.S.); pinicom-6@hotmail.co.jp (S.H.); tkmtsato@yokohama-cu.ac.jp (T.S.); hiro1017@yokohama-cu.ac.jp (K.H.); shin800m@yokohama-cu.ac.jp (S.K.); kubotak@yokohama-cu.ac.jp (K.K.); nrk20799@nifty.com (A.N.); 2 Department of Gastroenterology, Machida Municipal Hospital, Tokyo 194-0023, Japan; et310.polepole@gmail.com; 3 Department of Gastroenterology, Yokohama Rosai Hospital, Yokohama 222-0036, Japan; ysekino1978@gmail.com; 4 Department of Oncology, Yokohama City University Hospital, Yokohama 236-0004, Japan; norikoba@yokohama-cu.ac.jp; 5 Department of Gastroenterological Surgery, Yokohama City University Hospital, Yokohama 236-0004, Japan; endoit@yokohama-cu.ac.jp

**Keywords:** endoscopic ultrasound-guided fine needle aspiration, SEL, sampling adequacy rate, multivariate analysis

## Abstract

Endoscopic ultrasound-guided fine needle aspiration (EUS-FNA) is useful in diagnosing subepithelial lesions (SELs), and adequate tissue sampling is necessary to differentiate between benign and malignant diseases to determine therapeutic strategies. This study aimed to evaluate sampling adequacy and diagnostic performance of EUS-FNA for SELs with Franseen needles. This retrospective study enrolled 130 patients who underwent EUS-FNA with a 22-gauge needle for SELs from January 2010 to March 2021. We compared sampling adequacy and predictive factors influencing the sampling adequacy of EUS-FNA for SELs between Franseen and conventional needles. The sampling adequacy rates were 95.0% (38/40) with Franseen needles and 76.7% (69/90) with conventional needles (*p* = 0.011). The mean number of punctures with Franseen needles (2.80) was significantly less than that with conventional needles (3.42) (*p* < 0.001). In the multivariate analysis, the use of Franseen needles (*p* = 0.029; odds ratio [OR], 5.37; 95% confidence interval [CI], 1.18–23.36) was an independent factor influencing the sampling adequacy. Compared to conventional needles, the Franseen needle could play a vital role in accurately diagnosing SELs by yielding better sampling adequacy and reducing the number of passes.

## 1. Introduction

Subepithelial lesions (SELs) of the gastrointestinal tract are often encountered during endoscopy [1]. A gastrointestinal stromal tumor (GIST), which is an SEL with the potential to vary from benign to malignant, is found incidentally in endoscopy; thus, pathological evaluations of SELs are important to determine a treatment strategy [2,3,4]. It is crucial for endoscopists to make a differential diagnosis of SELs for potential malignancy, which can lead to further procedures such as resection. Since imaging detection sometimes results in an inaccurate diagnosis and endoscopic biopsy is challenging, it is essential for patients with SELs to receive adequate tissue sampling by endoscopic ultrasound-guided fine needle aspiration (EUS-FNA) [5,6].

**Figure 1:**
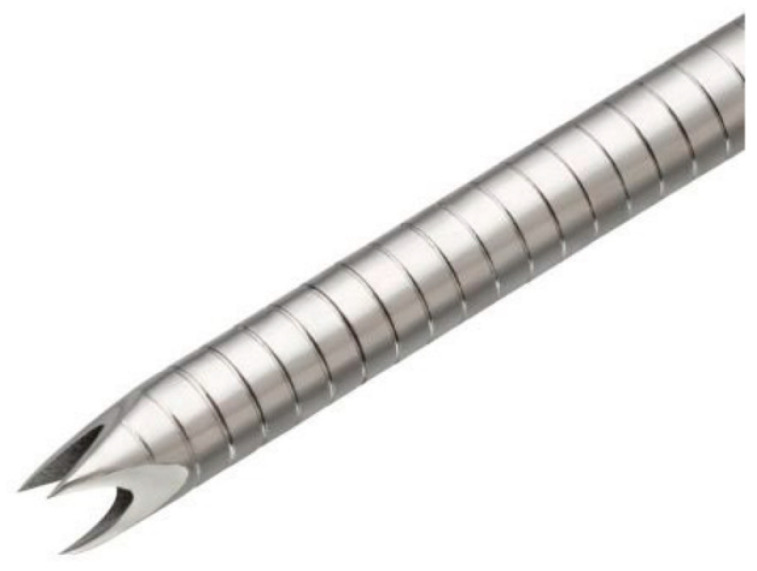
A Franseen needle, which has three points and heels on the tip. © 2022 Boston Scientific Corporation. All rights reserved. Reprinted with permission from [https://www.bostonscientific.com/pt-BR/produtos/agulhas/acquire.html]. 2022, Boston Scientific Corporation.

The sampling adequacy rate of EUS-FNA for SELs [7,8,9,10] has been reported, and EUS-FNA has been found to be useful for diagnosis. Various instrument innovations have been developed to enhance the sampling adequacy of EUS-FNA. The usefulness of a Franseen needle for EUS-FNA, which was developed with three novel, symmetric heels on the tip of the needle (Figure 1), has been established [7,11,12,13,14]. Although the sampling adequacy rate of the Franseen needle tends to be higher than that of the conventional needle in SELs <20 mm in diameter in a study with few cases [14,15], the superiority of Franseen needles for the sampling adequacy and diagnostic ability for SELs is also unclear [7]. There are no reports that clearly indicate that Franseen needles are an independent factor that contribute to sampling adequacy for SELs. Therefore, the present study aimed to compare the sampling adequacy rate, factors contributing to sampling adequacy, and diagnostic performance of EUS-FNA with the Franseen and conventional needles for SELs in the gastrointestinal tract.

## 2. Materials and Methods

### 2.1. Patients

This retrospective study involved 130 patients who underwent EUS-FNA with 22-gauge needles for SELs at Yokohama City University Hospital and Machida Municipal Hospital from January 2010 to March 2021.

### 2.2. EUS-FNA Indication and Procedure

In this study, EUS-FNA for SEL was performed in symptomatic cases and in cases with high-risk features such as ulceration, irregular borders, internal heterogeneity in EUS, and an increase in size during follow-up in accordance with the guidelines [4].

**Figure 2:**
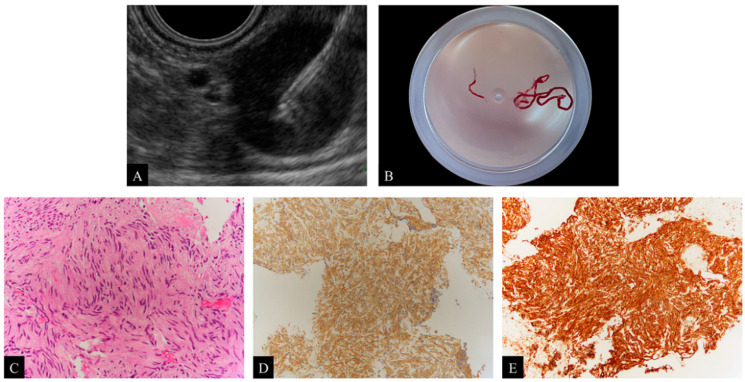
(**A**) Endoscopic ultrasound-guided fine needle aspiration (EUS-FNA) for subepithelial lesions (SELs) with the Franseen needle. (**B**) Visual confirmation of the presence of thin, whitish specimens after EUS-FNA with the Franseen needle. (**C**) Image showing a spindle cell tumor after hematoxylin and eosin (H&E) staining of the obtained specimens, ×200. (**D**) Immunohistochemical staining with c-kit is positive, ×100. (**E**) Immunohistochemical staining with CD34 is positive, ×100. Based on these pathological evaluations, the diagnosis is a gastrointestinal stromal tumor (GIST).

We intravenously administered 2–10 mg midazolam (Astellas, Tokyo, Japan), 5 mg diazepam (Takeda Pharma, Tokyo, Japan), and 15 mg pentazocine (Maruishi Pharma, Osaka, Japan) to patients to perform EUS-FNA with sedation. EUS-FNA procedures were performed with two different convex types of ultrasound endoscopes: a forward-oblique viewing endoscope (GF-UCT240 and GF-UCT260; Olympus Corporation, Tokyo, Japan) and a forward-viewing endoscope (TGF-UC260J; Olympus Corporation, Tokyo, Japan). These were connected to an ultrasound scanning system (EU-ME1 and EU-ME2 Premier Plus; Olympus Corporation, Tokyo, Japan). After the lesions were punctured, EUS-FNA was performed by stroking 20–30 times using negative suction with a 20-mL syringe (Figure 2A). These processes were carried out by experienced endoscopists who had performed more than 50 EUS-FNA procedures prior to this study. All EUS-FNA procedures were performed with 22-gauge needles. We used either a Franseen needle (Acquire; Boston Scientific Corporation, Tokyo, Japan) or conventional needles (EZ-Shot3 Plus; Olympus Corporation, Tokyo, Japan, EchoTip Ultra; Cook Medical Japan, Tokyo, Japan, Expect; Boston Scientific Corporation, Tokyo, Japan, SonoTip; Medico’s Hirata, Osaka, Japan). Institutions began using Franseen needles in October 2016. The definition of adverse events was established according to the severity grading system of the American Society for Gastrointestinal Endoscopy lexicon [16].

### 2.3. Histological Evaluation

The two institutions did not implement rapid on-site cytologic evaluation (ROSE) by cytopathologists or cytotechnologists. The EUS-FNA procedure was terminated after each endoscopist visually confirmed the presence of thin, whitish specimens (Figure 2B). The specimens were fixed in 10% formalin and embedded in paraffin. EUS-FNA specimens were stained with hematoxylin and eosin (H&E) for histologic examination (Figure 2C) and, if necessary for diagnosis, immunohistochemical staining was performed (Figure 2D,E). A diagnosis of mesenchymal tumors was made by comprehensive evaluation with immunohistochemical staining with c-kit, CD34, desmin, S-100 protein, and DOG-1. A positive c-kit staining with or without positive CD34 staining was diagnosed as GIST, positive desmin staining was diagnosed as leiomyoma, and positive S-100 staining was diagnosed as schwannoma. Immunohistochemical staining with other agents like cytokeratin, vimentin, Ki-67, and p53 was used to diagnose carcinoma or sarcoma, and staining with chromogranin A, synaptophysin, and CD56 was used to diagnose neuroendocrine neoplasm (NEN). Immunohistochemical staining was performed using an automated immunohistochemistry system (Ventana BenchMark ULTRA; Roche Diagnostics, Basel, Switzerland). The protocols are outlined as follows: for deparaffinization, the slides were heated to 72 °C; for antigen retrieval, the slides were heated to 95 °C and incubated for 8 min; the required antibodies were titrated onto the slides and incubated for 16 min, each diluted at 1:50 to 1:500, depending on the type of antibody. Experienced pathologists performed all analyses.

### 2.4. Definitions of Sampling Adequacy and Diagnostic Ability

Adequate sampling was defined as samples sufficient for histopathologic evaluations and immunohistochemical analyses.

The diagnostic ability of EUS-FNA was evaluated in terms of sensitivity, specificity, positive predictive value (PPV), negative predictive value (NPV), and accuracy. In this study of diagnostic ability, all surgery cases were included. Of the nonsurgical cases, those diagnosed adequately by EUS-FNA as malignant or benign with at least 6 months of clinical follow-up were included. Of the nonsurgical cases, those without sufficient EUS-FNA samples for histopathologic evaluation and immunohistochemical analysis and/or those without clinical follow-up for at least 6 months were excluded. The final diagnostic criteria were determined either by the surgical histopathological results of the cases of surgical resection or by EUS-FNA diagnosis and clinical follow-up of at least 6 months. The diagnostic ability of EUS-FNA was defined as follows: (1) the EUS-FNA histopathological examination was consistent with the diagnoses of the surgical resection specimens, and (2) the clinical follow-up course of at least 6 months without surgical resection corresponded to that of the EUS-FNA diagnoses.

We categorized the patients into two groups: (1) malignant group, including all GIST, carcinomas such as adenocarcinoma or squamous cell carcinoma, sarcoma, NEN, and malignant lymphoma, and (2) benign group, including leiomyoma, schwannoma, spindle cell tumor (containing mesenchymal tumors for which immunostaining was not performed), aberrant pancreas, inflammatory granuloma, lymph node, hematoma, mucosal prolapse syndrome, gastritis, and lipoma. Non-diagnosis cases were defined as cases excluded from this study of diagnostic ability, nonsurgical cases without adequate sampling of EUS-FNA, and/or cases without clinical follow-up for at least 6 months.

### 2.5. Factors Influencing the Sampling Adequacy of EUS-FNA

Variables employed for univariate and multivariate analysis were gender (females vs. males), tumor size (<20 mm vs. ≥20 mm), location of the lesion (esophagus, stomach, duodenum, and rectum), EUS-FNA procedure period (2017–2021 vs. 2010–2016), needle shape (Franseen needle vs. conventional needle), and the endoscope (forward-viewing ultrasound endoscope: TGF-UC260J vs. forward-oblique viewing ultrasound endoscope: GF-UCT240 and GF-UCT260).

### 2.6. Endpoints

The primary endpoint was the sampling adequacy rate of EUS-FNA for SELs using Franseen and conventional needles. Secondary endpoints were factors influencing the sampling adequacy rate. We also assessed the sensitivity, specificity, PPV, NPV, and accuracy of EUS-FNA for SELs.

### 2.7. Statistical Analysis

Continuous variables were presented as median (range) or mean ± standard deviation. Statistical analysis was conducted using the chi-squared test (or Fisher’s exact, if appropriate) for categorical variables and the Student’s *t*-test or Mann–Whitney U test for continuous variables for univariate analyses. The Student’s *t*-test was used if a normal distribution was likely, and the Mann–Whitney U test was used if normality could not be demonstrated. Logistic regression analysis was used for multivariate analysis. Values of *p* < 0.05 were regarded as statistically significant. Multivariable analysis was performed by selecting variables with *p* < 0.05 in univariate analysis. SPSS version 28 software (IBM Corp, Armonk, NY, USA) was used for statistical analysis.

## 3. Results

### 3.1. Patient and Lesion Characteristics

All 130 patients who underwent EUS-FNA for SELs at the two institutions were enrolled. The patient and lesion characteristics of EUS-FNA for SELs are shown in Table 1. The median age of the patients was 65.0 years (range, 23–90 years), 46.2% (60/130) were females, and the median diameter of the SELs was 25.0 mm (range, 8.0–90.5 mm). Tumor locations were the stomach in 96 cases (73.8%), the duodenum in 21 cases (16.2%), the rectum in 10 cases (7.7%), and the esophagus in 3 cases (2.3%). Patient and lesion characteristics showed no significant difference between the Franseen and conventional needles.

Among 130 cases, 72 (55.4%) were surgically resected. The final diagnosis of all SELs for which EUS-FNA was performed is shown in Table 2. The most frequent diseases were GIST (52.3%, 68/130), followed by leiomyoma (7.7%, 10/130), carcinoma (7.7%, 10/130), and schwannoma (4.6%, 6/130). All non-diagnosis cases were nonsurgical; 17 cases did not have adequate sampling for EUS-FNA, and 2 cases were not followed-up for more than 6 months.

### 3.2. Sampling Adequacy Rates and Outcomes

The comparison of the outcomes of EUS-FNA performed with either Franseen or conventional needles is shown in Table 3. The puncture technique was successful in all cases (130/130). The overall sampling adequacy rate of EUS-FNA for SELs was 82.3% (107/130). The Franseen needles had a significantly superior sampling adequacy rate compared to conventional needles (95.0% vs. 76.7%; *p* = 0.011). Endoscopists visually confirmed the presence of thin, whitish specimens, which could be attained with one to six punctures. The mean overall number of punctures was 3.23 ± 0.95, and the mean number of punctures for Franseen needles was significantly lower than that of conventional needles (2.80 ± 0.87 vs. 3.42 ± 0.92; *p* < 0.001). Two cases (1.5%; 2/130) experienced bleeding adverse events with conventional needles, but none of them required blood transfusions. There were no deaths or serious adverse events related to EUS-FNA in this study.

### 3.3. Analysis of Factors influencing the Sampling Adequacy

Univariate and multivariate analyses were performed to identify the factors influencing the sampling adequacy rate of EUS-FNA for SELs (Table 4). Univariate analysis revealed duodenal lesions (*p* = 0.047) and the use of Franseen needles (*p* = 0.011) as significant factors. However, gender, tumor size, procedure period, and type of endoscope were found to be non-significant factors. In multivariate analysis, the use of Franseen needles (*p* = 0.029; odds ratio [OR], 5.37; 95% confidence interval [CI], 1.18–23.36) was an independent factor influencing the sampling adequacy of EUS-FNA for SELs.

### 3.4. Comparison of Diagnostic Ability

The diagnostic ability was studied in 111 cases, including all 72 surgical cases and 39 nonsurgical cases excluded without adequate sampling of EUS-FNA and/or without clinical follow-up for at least 6 months. The overall sensitivity, specificity, PPV, NPV, and accuracy of EUS-FNA for SELs were 96.4% (81/84), 96.3% (26/27), 98.8% (81/82), 89.7% (26/29), and 96.4% (107/111), respectively, as shown in Table 5. Comparing Franseen and conventional needles, the accuracy was higher for Franseen needles (100% vs. 94.7%; *p* = 0.302), but there was no significant difference.

## 4. Discussion

The current study conducted in the two institutions showed that the Franseen needle, as an independent factor in multivariate analysis, was associated with a better sampling adequacy rate than the conventional needle. For needle penetration into the tissue, a smaller included angle of the cutting edge radius and a smaller inclination angle of cutting edge relative to the cutting direction are advantageous because they reduce the insertion force. As a result, the Franseen needle, with a symmetrical three-heeled geometry, reduces the included and inclination angles and enables easier and more secure tissue acquisitions than the lancet shape of the conventional needle [17]. Regarding EUS-FNA for SELs, the Franseen needle would be preferable for endoscopists in terms of sampling adequacy and high diagnostic ability compared to the conventional needle. The lack of a significant difference in the accuracy between Franseen and conventional needles may be because many cases were excluded from the diagnostic ability study due to inadequate sampling adequacy with the conventional needle.

Furthermore, this study showed that the Franseen needle could reduce unnecessary punctures. Although the use of Franseen needles has been shown to reduce the number of punctures in pancreatic lesions [7,18], there have been no such clear reports for SELs, which was novel in this study. Despite the few punctures, the Franseen needle provided a sufficient number of samples for pathology in the present study. As a result, the Franseen needle yields sufficient specimens to enable visual assessment by endoscopists.

ROSE is an effective method to reduce the number of EUS-FNA punctures [19,20], but not all endoscopic units can perform ROSE due to lack of manpower; in this study, ROSE was not performed. Evidence demonstrating that the Franseen needle reduces the number of EUS-FNA punctures for SELs, as in the present study, might be valuable for endoscopic units where ROSE is unavailable.

EZ-shot3 (Olympus Corporation, Tokyo, Japan), EchoTip Ultra (Cook Medical Japan, Tokyo, Japan), Expect (Boston Scientific Corporation, Tokyo, Japan), and SonoTip (Medico’s Hirata, Osaka, Japan), which were used as the conventional needles, were respectively priced at JPY 27,000, JPY 27,000, JPY 27,000, and JPY 26,000, while Acquire (Boston Scientific Corporation, Tokyo, Japan), which was used as the Franseen needle in this study, was priced at JPY 32,000. The Franseen needle was more expensive than conventional needles; however, the sampling adequacy rate of conventional needles was 76.7%, and the sampling adequacy rate of the Franseen needle was 95.0% in this study. The sampling adequacy rate of conventional needles was 18.3% lower than that of the Franseen needle and is therefore associated with the risk of increasing the number of patients who need to perform a repeat EUS-FNA, thus increasing the medical costs. Therefore, using Franseen needles in EUS-FNA for SELs may reduce total costs compared to using conventional needles.

In univariate analysis, the use of a Franseen needle and a duodenal lesion were factors affecting the sampling adequacy rate of EUS-FNA for SELs. There was no significant difference in patient age, gender, tumor size, or scope type. The size of the SEL did not affect the sampling adequacy in our study, indicating that EUS-FNA for SELs with a Franseen needle, if successful, provides sampling adequacy whether or not the tumor diameter was <20 mm. The significantly lower sampling adequacy rate for duodenal lesions compared to other gastrointestinal tracts may be related to the fact that puncture resistance is very high in positions that require an upward EUS angle, as described by Itoi et al. [21].

The impact of the Franseen needle, which was mainly used in this study, has been shown for SELs [7,14], and other new needles, such as needles with side holes [22] and fork-tips [23,24], have been developed and show a good sampling adequacy rate for SELs. Despite this increase in the number of puncture needle options, there is no one particular needle used uniformly. Regarding needle selection, needle size has also been discussed; needle size may be a confounder, as the potential usefulness of 19-gauge [25,26,27] and 25-gauge [28] puncture needles has been shown. Although a 19-gauge needle can provide sufficient tissue, the strong puncture resistance makes it very difficult to use a 19-gauge needle, especially for small lesions. Furthermore, the specimens may be too small for a 25-gauge needle. Therefore, 22-gauge needles are more commonly used in EUS-FNA. In the present study, only 22-gauge puncture needles were included, and a 22-gauge Franseen needle showed a good sample collection rate. In the case of SELs, a 22-gauge Franseen needle is considered sufficient to obtain sufficient samples for cell block preparation and subsequent immunohistochemical staining, which is very useful for diagnosing SEL subtypes, and sampling of these lesions should be done routinely.

Recently, EUS-FNA has been shown to have utility not only for diagnosing lesions but also for genomic characterization to develop molecular profiling to find personalized anti-cancer therapies [29]. Genomic characterization for GIST is also attracting attention [30,31], but surgical specimens are often used since genomic characterization requires sufficient tissue sampling. The Franseen needle, with a high sampling adequacy rate, may have a role in the potential genomic characterization for GIST. Consequently, the Franseen needle would meet future medical needs.

We had several limitations in this study: first, the retrospective nature of the study had inherent limitations; second, one type of Franseen needle was compared to several types of conventional needles in this study; and third, the current study included the possibility of needle selection bias by the endoscopists.

## 5. Conclusions

While the sampling adequacy rate for duodenal lesions was significantly lower and should be treated with caution, the Franseen needle was also an independent factor in improving the sampling adequacy rate of EUS-FNA for SELs. EUS-FNA for SELs with the Franseen needle had a significantly better sampling adequacy rate with fewer punctures than the conventional needles.

## Figures and Tables

**Table 1:** Patient and lesion characteristics of cases with EUS-FNA for SELs (*n* = 130).

Variables	Total	Franseen	Conventional	*p*-Value
	*n* = 130	*n* = 40	*n* = 90	
Age (years)				0.772
Median (range)	65.0 (23–90)	65.0 (44–84)	64.5 (23–90)	
Gender, *n* (%)				0.348
Females	60 (46.2)	16 (40.0)	44 (48.9)	
Males	70 (53.8)	24 (60.0)	46 (51.1)	
Lesion size (mm)				0.739
Median (range)	25.0 (8.0–90.5)	24.0 (10.0–80.0)	25.0 (8.0–90.5)	
Lesion location, *n* (%)				0.145
Esophagus	3 (2.3)	1 (2.5)	2 (2.2)	
Stomach	96 (73.8)	29 (72.5)	67 (74.4)	
Duodenum	21 (16.2)	4 (10.0)	17 (18.9)	
Rectum	10 (7.7)	6 (15.0)	4 (4.4)	

EUS-FNA, endoscopic ultrasound-guided fine needle aspiration; SELs, subepithelial lesions.

**Table 2:** Details of final diagnosis of all SELs (*n* = 130).

Disease, *n* (%)	No.		
	Total	Surgical	Nonsurgical
	*n* = 130	*n* = 72	*n* = 58
GIST	68 (52.3)	55 (76.4)	13 (22.4)
Leiomyoma	10 (7.7)	3 (4.2)	7 (12.1)
Carcinoma	10 (7.7)	5 (6.9)	5 (8.6)
Schwannoma	6 (4.6)	3 (4.2)	3 (5.2)
Sarcoma	3 (2.3)	0	3 (5.2)
NEN	2 (1.5)	1 (1.4)	1 (1.7)
Aberrant pancreas	2 (1.5)	0	2 (3.4)
Hematoma	2 (1.5)	2 (2.8)	0
Lymph node	2 (1.5)	0	2 (3.4)
Inflammatory granuloma	1 (0.8)	1 (1.4)	0
MALT lymphoma	1 (0.8)	0	1 (1.7)
MPS	1 (0.8)	0	1 (1.7)
Gastritis	1 (0.8)	0	1 (1.7)
Hyperplasia	1 (0.8)	1 (1.4)	0
Lipoma	1 (0.8)	1 (1.4)	0
Non-diagnosis	19 (14.6)	0	19 (32.8)

SELs, subepithelial lesions; GIST, gastrointestinal stromal tumor; NEN, neuroendocrine neoplasm; MALT lymphoma, mucosa-associated lymphoid tissue lymphoma; MPS, mucosal prolapse syndrome.

**Table 3:** Comparison of technical EUS-FNA outcomes for SELs (*n* = 130).

Variables	Total	Franseen	Conventional	*p*-Value
	*n* = 130	*n* = 40	*n* = 90	
Puncture success	100% (130/130)	100% (40/40)	100% (90/90)	N.S.
Adverse events				
Bleeding	1.5% (2/130)	0	2.2% (2/90)	1.000
Perforation	0	0	0	N.S.
Infection	0	0	0	N.S.
Death	0	0	0	N.S.
Number of punctures				
Mean ± SD	3.23 ± 0.95	2.80 ± 0.87	3.42 ± 0.92	<0.001
Sampling adequacy rate	82.3% (107/130)	95.0% (38/40)	76.7% (69/90)	0.011

EUS-FNA, endoscopic ultrasound-guided fine needle aspiration; SELs, subepithelial lesions; SD, standard deviation; N.S., not significant.

**Table 4:** Factors influencing the sampling adequacy of EUS-FNA for SELs (*n* = 130).

Factors	Sampling Adequacy	Univariate Analysis	Multivariate Analysis		
		*p*-Value	OR	95% CI	*p*-Value
Gender					
Females	83.3% (50/60)	0.777	−	−	−
Males	81.4% (57/70)				
Tumor size					
<20 mm	73.3% (22/30)	0.142	−	−	−
≥20 mm	85.0% (85/100)				
Location					
Esophagus	66.7% (2/3)	0.485			
Stomach	85.4% (82/96)	0.124			
Duodenum	63.6% (14/22)	0.047	0.39	0.13–1.15	0.088
Rectum	90.0% (9/10)	0.515			
Period					
2010–2016	78.4% (58/74)	0.177	−	−	−
2017–2021	87.5% (49/56)				
Shape of needle					
Franseen	95.0% (38/40)	0.011	5.37	1.18–23.36	0.029
Conventional	76.7% (69/90)				
Field of view					
Forward	100% (7/7)	0.352	−	−	−
Forward-oblique	81.3% (100/123)				

EUS-FNA, endoscopic ultrasound-guided fine needle aspiration; SELs, subepithelial lesions; OR, odds ratio; CI, confidence interval; −, not analyzed.

**Table 5:** Diagnostic ability of EUS-FNA for SELs compared with Franseen and conventional needles (*n* = 111).

		Needle		
	Total	Franseen	Conventional	
Cases (*n*)	*n* = 111	*n* = 36	*n* = 84	*p*-Value
Sensitivity	96.4% (81/84)	100% (29/29)	94.5% (52/55)	0.548
Specificity	96.3% (26/27)	100% (7/7)	95.0% (19/20)	1.000
PPV	98.8% (81/82)	100% (29/29)	98.1% (52/53)	1.000
NPV	89.7% (26/29)	100% (7/7)	86.4% (19/22)	0.557
Accuracy	96.4% (107/111)	100% (36/36)	94.7% (71/75)	0.302

EUS-FNA, endoscopic ultrasound-guided fine needle aspiration; SELs, subepithelial lesions; PPV, positive predictive value; NPV, negative predictive value.

## Data Availability

Data available on request because of restrictions, e.g., privacy or ethics.
